# Differential importance of nucleus accumbens Ox1Rs and AMPARs for female and male mouse binge alcohol drinking

**DOI:** 10.1038/s41598-020-79935-2

**Published:** 2021-01-08

**Authors:** Claudina Kwok, Kelly Lei, Vincent Pedrozo, Lexy Anderson, Shahbaj Ghotra, Margaret Walsh, Laura Li, JiHwan Yu, Frederic Woodward Hopf

**Affiliations:** 1 California State University East Bay, Hayward, CA USA; 2 Department of Neurology, University of California at San Francisco, San Francisco, CA USA; 3 Department of Psychiatry, Indiana University School of Medicine, 320 W. 15th Street, NB 300E, Indianapolis, IN 46202 USA

**Keywords:** Motivation, Reward

## Abstract

Alcohol use disorder exhausts substantial social and economic costs, with recent dramatic increases in female problem drinking. Thus, it is critically important to understand signaling differences underlying alcohol consumption across the sexes. Orexin-1 receptors (Ox1Rs) can strongly promote motivated behavior, and we previously identified Ox1Rs within nucleus accumbens shell (shell) as crucial for driving binge intake in higher-drinking male mice. Here, shell Ox1R inhibition did not alter female mouse alcohol drinking, unlike in males. Also, lower dose systemic Ox1R inhibition reduced compulsion-like alcohol intake in both sexes, indicating that female Ox1Rs can drive some aspects of pathological consumption, and higher doses of systemic Ox1R inhibition (which might have more off-target effects) reduced binge drinking in both sexes. In contrast to shell Ox1Rs, inhibiting shell calcium-permeable AMPA receptors (CP-AMPARs) strongly reduced alcohol drinking in both sexes, which was specific to alcohol since this did not reduce saccharin intake in either sex. Our results together suggest that the shell critically regulates binge drinking in both sexes, with shell CP-AMPARs supporting intake in both sexes, while shell Ox1Rs drove drinking only in males. Our findings provide important new information about sex-specific and -general mechanisms that promote binge alcohol intake and possible targeted therapeutic interventions.

## Introduction

Despite extensive efforts, alcohol use disorder (AUD) remains a significant health problem, with substantial medical, social and economic costs^1–6^. Binge drinking, with high levels of alcohol intake, is an especially problematic and pernicious obstacle to treating AUD. About 3/4th of AUD-related costs are due to individuals who binge^6^, and reducing binge intake lowers health risks^3,5^ and relapse^1^. Also, binging in non-dependent, problem drinkers can promote development of more serious alcohol problems^7–9^. Thus, identifying key mechanisms that drive alcohol binge intake may provide novel and translationally useful therapeutic interventions and reduce the burden of alcohol-related costs, especially given the limited pharmacotherapies for AUD^10^.

In recent years, the rate of hazardous alcohol drinking in human females has risen dramatically^11–13^, making it essential to understand possible mechanistic differences across the sexes that promote binge intake. Sex differences are known to exist for several addiction-related behaviors^14^, including where female rodents often drink more alcohol than males^14,15^. Other aspects of alcohol responding have some divergence. For example compulsion-like responding, where intake persists despite negative consequences^16,17^, is more similar between sexes in some models^15,18^, including home-cage limited-access drinking^15^ used here, but not others^19,20^ (see also “Discussion”).

In seeking the critical mechanisms that drive binge drinking, we have focused on the nucleus accumbens (NAcb) shell (shell) and the contribution of orexin-1-receptors (Ox1Rs) and AMPA-type glutamate receptors (AMPARs) (reviewed in^21,22^). The shell is a critical regulator of numerous motivated and addiction-related behaviors^22–25^ and compulsion-like behaviors^26–28^, including where shell inhibition reduces alcohol drinking but not sweet fluid intake or locomotor activity^29–36^. Orexin/hypocretins regulate many adaptive behavioral and physiological responses^37,38^, and Ox1Rs are of particular interest because they drive responding for high-value natural and drug rewards, including alcohol, with little role for less-motivating substances^37–42^. Indeed, shell Ox1R inhibition decreases binge intake in males, especially higher drinkers^29,43^ (as seen with systemic block of Ox1Rs^44–46^), and systemic Ox1R inhibition reduces many forms of pathological alcohol intake^38,44–50^. In addition, calcium-permeable AMPARs (CP-AMPARs) are observed in the shell after exposure to alcohol^51–53^ and other intoxicants and stress-related conditions (see^22^). In addition, shell CP-AMPARs are known to promote several addiction-related behaviors (reviewed in^22^), making it imperative to know whether CP-AMPARs in shell are also important modulators of alcohol binge consumption.

Here, we demonstrate that shell Ox1Rs did not regulate binge alcohol drinking in female C57BL/6 mice, in strong contrast to our previously demonstrated Ox1R regulation of binge intake in male mice^29,43^. However, lower doses of systemic Ox1R inhibition did reduce compulsion-like alcohol drinking in females, similar to males^48^, indicating that females have Ox1Rs that can regulate some aspects of pathological alcohol-directed behavior. Furthermore, inhibition of CP-AMPARs in the shell significantly reduced alcohol drinking in both males and females. This was specific for alcohol, as inhibiting shell CP-AMPARs had no impact on saccharin intake. Thus, binge alcohol consumption in females required shell CP-AMPARs, similar to males, but did not involve shell Ox1Rs, very different from males. These findings indicate that complementary but partly separable mechanisms in the shell drive binge alcohol drinking in females versus males, with important implications for differential treatment strategies to counteract alcohol addiction in the sexes.

## Material and methods

### Limited daily access (LDA) drinking

All procedures followed Guide for Care and Use of Laboratory Animals provided by the NIH, with approval of the UCSF IACUC. Single housed adult C57BL6/J mice (Jackson Labs) drank under a limited daily access (LDA) paradigm, which is a two-bottle choice (2BC) variant of Drinking-in-the-Dark (with 15% alcohol (v/v) versus water), as we^29,43,48,54^ (see also^55^) have used previously. At 7–8 weeks of age, mice had a single 24-h 2BC session. Thereafter, mice drank under LDA for 2 h/day, 5 days/week, starting 2.5–3 h into the dark cycle. After ~ 3 week LDA, with handling 2–5 min per day for the week before surgery, mouse cohorts intended for microinjections underwent intra-shell cannulation surgery (details below). After 1-wk recovery and ~ 2-week more LDA, there was a week of LDA with handling: 2–3 days of handling (2–5 min/day), then 2–3 days of handling where the cannula plug was removed and returned, then 1 day with saline injection. Thereafter, we began intracranial injection experiments (details below). These methods and those below were the same as those used in our previous studies of shell Ox1Rs in male mice^29,43,48,54^. Systemic injection studies occurred with equivalent timing except without surgery. Separate cohorts of mice were used for each experiment, including systemic injections.

Quinine-resistant alcohol drinking was tested by adulterating alcohol with 100 µM quinine, as in previous studies^15,48^. For saccharin drinking (tested in separate cohorts of mice), the timing and length of the session across days was the same as for alcohol, except mice instead consumed 0.05% saccharin. We have previously used this^29,43,48^ since mice drink approximately the same volume of this concentration of saccharin as with alcohol. Blood alcohol concentrations were determined exactly as previously described^48^.

To compare basal drinking levels (determined on vehicle test days) with possible changes in drinking with a pharmacological agent, we used a method as in^43^. In particular, determining the percent change in drinking after drug exposure (100 × (drug/vehicle) – 100) can result in large difference when drinking levels under vehicle are lower. Thus, we instead used a method involving a log transformation of the change in drinking with drug, log(100 × drug/vehicle). The strengths and weaknesses of these two methods (percent change vs log transformed percent change) are discussed in detail in^43^.

### Surgery and microinjection

Intra-shell cannulation methods were as in^29,43^. Surgery occurred after 3-week LDA, with 1-wk recovery before resuming LDA. Cannula were implanted targeting shell (AP + 1.5 mm, ML ± 0.5 mm, DV − 4.5 mm). Pharmacological agents were microinjected (0.2 µl/side, 33-gauge needle extending 0.3 mm beyond cannula tip, at a rate of 200nL/min) 30 min before a drinking session. Vehicle or receptor blocker was administered 1-week apart, using a within-subject, Latin-squares, randomized design. Importantly, animals were exposed to each experimental condition twice: conditions (drug vs vehicle) were randomized for one round of injections, then the same schedule was used for a second round. Each animal thus received 5–6 injections, where 5 injections were planned (initial saline during handling and 4 experimental injections) and a 6th occurred if there were problems with injector clogging (which happened infrequently). For each experimental condition from a given animal, drinking data from the two injection days were averaged to give a single intake value for vehicle and a single value for drug. We routinely utilize this approach to reduce variability in drinking measures^29,43,48,56,57^. Histology was performed to verify the location of cannula placements, and is shown in Suppl. Fig. 1.

### Reagents

All intracranial reagents were injected bilaterally in 0.2 µl/side (see above). We utilized SB-334867 (SB) as an Ox1R inhibitor^29,48^ since it has been used across many studies, with well-established dosages and specific behavioral effects^37,38,40,42,58–61^. Also, behaviors inhibited by SB are reduced by other Ox1R inhibitors^38^. The SB dose used intracranially (3 µg/side) reduces alcohol but not saccharin intake^29^ without effect in the NAcb Core, nor in the shell at 1 µg/side^29,43^, and Ox2R inhibition in the shell with an equivalent dose of TCS-OX2-29 has no effect on male binge alcohol intake^29^. This SB dose also decreases stress but not drug-prime reinstatement of morphine CPP, or locomotor activity, when infused in the shell^61^, reduces alcohol- but not sucrose-seeking (mPFC^58^) and decreases alcohol but not saccharin or food intake (icv^60^). Thus, these findings validate SB as an Ox1R inhibitor with selective impacts on reward-directed behavior. It also would be useful, in future studies, to identify how well systemic SB crosses the blood–brain barrier, the mechanisms of systemic SB first-pass metabolism, and the spread of SB after intracranial injection.

Calcium-permeable AMPARs (CP-AMPARs) were inhibited using 1-naphthylacetyl spermine (NASPM) at 20 µg/side, as used in many studies^62–65^. This and higher doses in NAcb decrease cocaine seeking, but have no effect on cocaine self-administration, sucrose seeking^62,65^ or behavioral flexibility^66^.

SB and NASPM were purchased from Sigma. Drugs were made fresh for each day of use. NASPM was diluted in 0.9% sterile saline (NaCl) for intracranial injections. For systemic injections, SB at 3 mg/kg was diluted in 2% DMSO and 25% beta-Cyclodextrin (BCD) and SB at 30 mg/kg was diluted in 2% Tween 80 and saline. For intracranial injections, SB was diluted in 100% DMSO: while 100% DMSO is a high dose for intracranial, we^29,43^ and other groups^67–70^ have used this intracranial vehicle and shown that it does not have non-specific effects. Importantly, our studies are performed with a randomized, Latin-squares design, with alcohol drinking on days in between intracranial test sessions. Any possible lingering toxicity of DMSO should impact drinking on subsequent days, but this was not observed (see^29^). However, others have reported the possibility of DMSO-related damage^71,72^, and thus, despite within-animal comparisons used here and in our other studies, we cannot rule out the possibility of DMSO-related changes in animals studied here.

### Statistics and analyses

The majority of comparisons were within-animal (vehicle vs drug). These were examined using paired t-test for normally distributed data and Wilcoxon matched-pair signed rank test (Wilcoxon) for non-normal data, and between-animal comparisons were examined using t-test, or Mann–Whitney test for non-normal data. We also performed two-way ANOVA with sex between-factor and vehicle/drug within-factor for all conditions where t-tests showed significant differences. In some comparisons, subject’s data were normalized to baseline to compare across sexes. We also analyzed the correlation between basal alcohol drinking levels and impact of a given treatment (determined as log [100 × (intake during drug treatment)/(intake during vehicle)], as in^43^, where log value of 2 (log [100]) indicates no treatment effect). Statistical comparisons were performed with GraphPad Prism or SPSS. Data are shown as mean ± SEM and scatter of raw data, and, for comparison, box-and-whiskers plots in grey, with bars showing 25%-75% range, whiskers showing 5%-95% range, and median shown by crossbar; some data point values are given in figure legend when much higher than other data.

## Results

In agreement with previous findings^14,15^, female alcohol-only drinking was significantly higher (3.33 ± 0.17 g/kg) than male intake (2.38 ± 0.13 g/kg) (*t*_55_ = 3.881, *p* = 0.0003). In contrast, quinine-resistant consumption did not differ between sexes (females: 1.97 ± 0.21 g/kg; males: 1.99 ± 0.18 g/kg; *t*_44_ = 0.0780, *p* = 0.9369). Both were measured in the week before intracranial injections. Also, in separate groups of mice, we examined blood alcohol concentrations (BACs) achieved in male and female alcohol-drinking mice after a 2 h intake session. BACs strongly correlated with intake in both sexes (Suppl. Fig. 2). Females (*n* = 12) had an average of 4.02 ± 0.29 g/kg and 107.96 ± 15.62 mg% BAC, with correlation of *p* = 0.0473 (R^2^ = 0.3383). Males (*n* = 10) had an average of 2.68 ± 0.32 g/kg and 99.72 ± 23.44 mg% BAC, with correlation of *p* = 0.0002 (R^2^ = 0.8330). Thus, considering 80 mg% to reflect binge-level intake, both sexes on average showed binge-level consumption levels under the LDA alcohol-only intake model used here.

**Figure 1:**
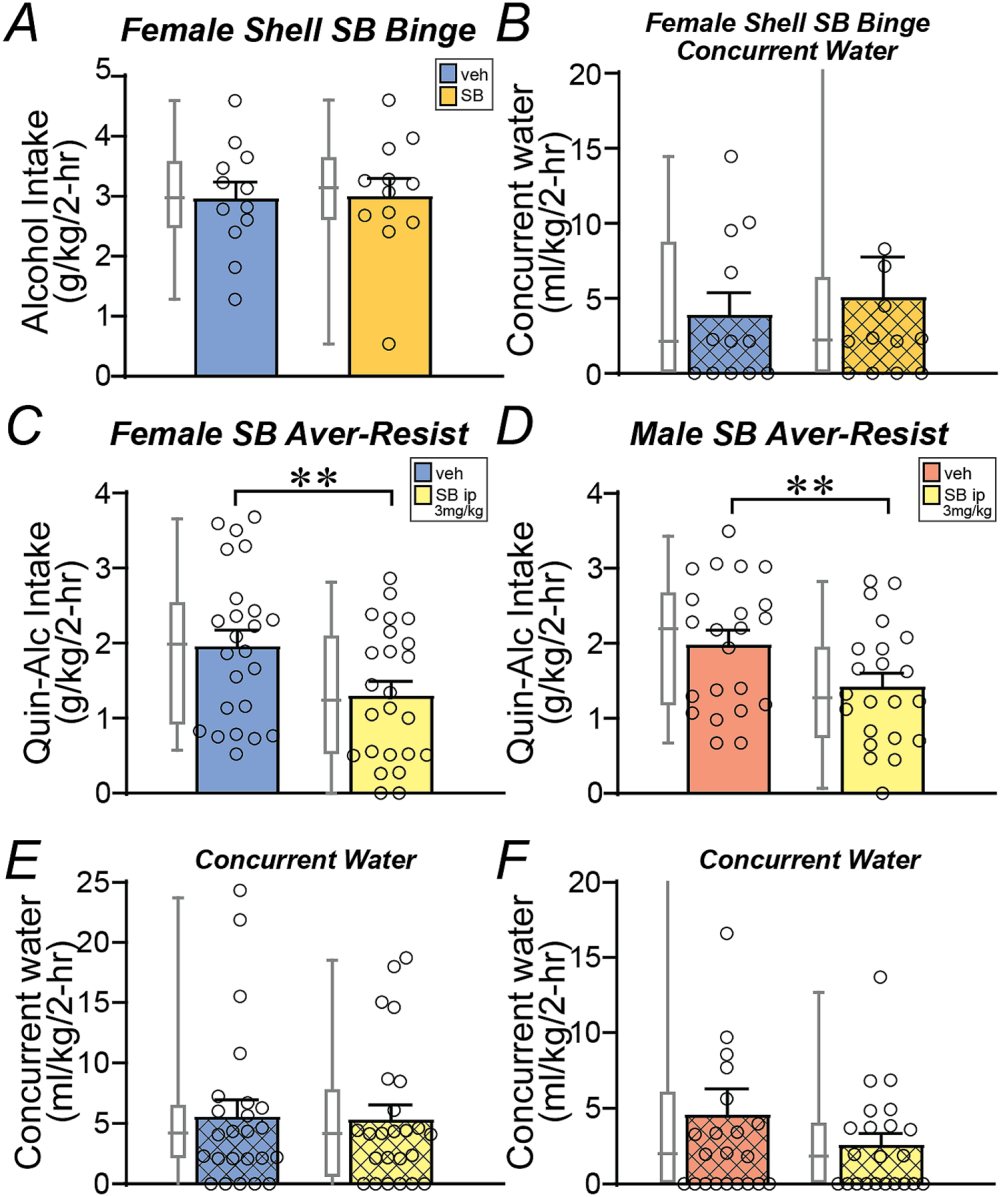
Ox1R inhibition and regulation of binge and compulsion-like drinking in female C57 mice. (**A**,**B**) Intra-shell infusion of the Ox1R inhibitor SB did not alter (**A**) alcohol-only consumption under LDA (2-h per day drinking), nor (**B**) did it reduce concurrent water intake. (**C**,**D**) Systemic administration of a lower dose of SB (3 mg/kg) significantly reduced quinine-resistant alcohol drinking in both (**C**) females and (**D**) males. (**E**,**F**) However, lower dose SB did not significantly reduce concurrent water intake in either sex, suggesting a specific effect of systemic SB on compulsion-like alcohol consumption. One data point in (**B**) for SB was 32.7 ml/kg and in (**F**) for vehicle was 33.6 ml/kg, not shown on graphs. For Figs. 1, 2, 3 and 4, box-whisker plot (see “Material and methods”) is for the same data shown in adjacent colored bar. Aver-Resist: aversion-resistant drinking; Quin-Alc: quinine (100 µM) in alcohol. ** *p* < 0.01.

We have previously shown that shell Ox1Rs are critical for promoting binge alcohol drinking in male C57BL6/J mice, especially in higher-drinking subjects^29,43^, using the broadly utilized Ox1R blocker SB-334867 (SB) at a dose from many other studies^29,58,61,73–75^ (see “Material and methods”). Thus, we examined the impact of this SB dose in the shell on alcohol-only intake in female C57BL6/J mice. Interestingly, and unlike in males^29,43^, SB inhibition of Ox1Rs in the shell did not alter alcohol-only intake in females (Fig. 1A, *n* = 12, *t*_11_ = 0.1372, *p* = 0.8934), nor did it alter concurrent water intake (Fig. 1B, *p* = 0.8457, Wilcoxon). A two-way ANOVA found a significant sex-treatment interaction (F_1,41_ = 4.516, *p* = 0.040) but no overall treatment effect (F_1,41_ = 3.751, *p* = 0.060); data for male intra-shell SB binge drinking (*n* = 31) were from Lei et al.^43^, and the trend for treatment effect may reflect the larger sample size in males vs females.

One possibility is that female alcohol intake might more generally occur without the need for Ox1Rs. Thus, we next examined whether quinine-resistant alcohol intake might be inhibited by an Ox1R blocker; we previously showed that a lower dose of SB (3 mg/kg, i.p.), which is low enough to assure specificity for Ox1Rs (see^21^), reduces quinine-resistant but not alcohol-only intake in male mice^48^. Also, male and female mouse quinine-resistant drinking under limited access shows similar sensitivity to quinine in alcohol^15^. Consistent with our previous findings in males^48^, quinine-resistant alcohol consumption was significantly reduced by systemic injection of 3 mg/kg SB in both females (Fig. 1C, *n* = 24, *t*_23_ = 0.4.524, *p* = 0.0002) and males (Fig. 1D, *n* = 22, *t*_21_ = 0.2.792, *p* = 0.0109), with a similar reduction in drinking across the sexes (Mann–Whitney *p* = 0.4816). A two-way ANOVA found a significant effect of treatment (F_1,44_ = 24.791, *p* < 0.001) but no sex-treatment interaction (F_1,22_ = 0.168, *p* = 0.684). These results suggest that, even though shell Ox1Rs did not regulate female alcohol-only intake, Ox1Rs can play a role in regulating at least some forms of alcohol drinking in females. In addition, 3 mg/kg systemic SB did not significantly alter concurrent water consumption during compulsion-like intake in females (Fig. 1E, *p* = 0.8695, Wilcoxon) nor males (Fig. 1F, *p* = 0.6473, Wilcoxon), indicating a specific effect of systemic SB on aversion-resistant alcohol intake rather than consumption more generally. The trend apparent in Fig. 1F is not significant, and our previous studies show no effect of 3 mg/kg SB on concurrent water drinking during compulsion-like drinking^48^. Taken together, these findings indicate that some forms of alcohol drinking (aversion-resistant intake) can require Ox1Rs in both females and males, but that shell Ox1Rs were not needed for female binge alcohol-only drinking, unlike in males^29,43^.

**Figure 2:**
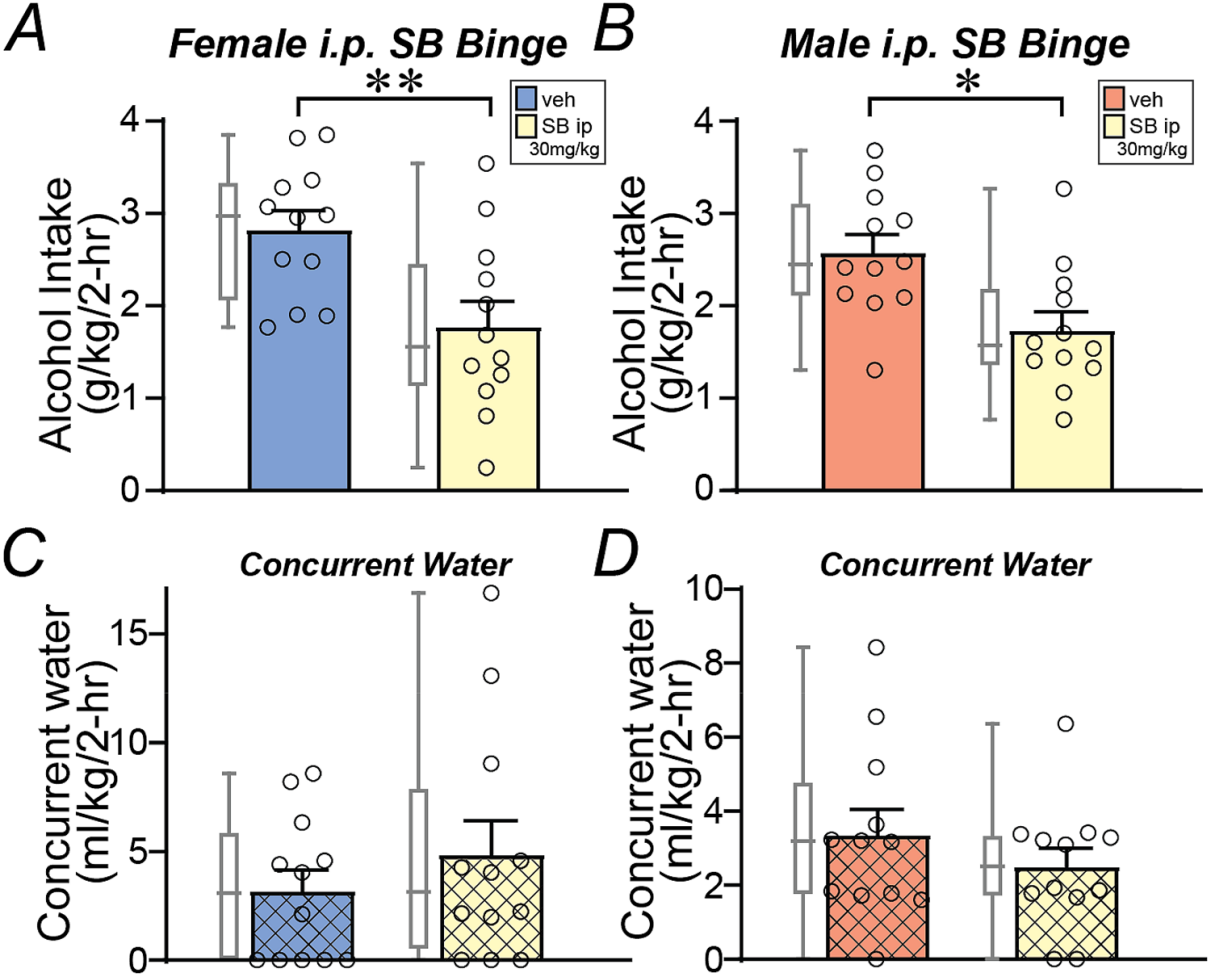
Higher doses of systemic Ox1R inhibitor reduced alcohol-only drinking in both sexes. (**A**,**B**) Higher dose SB (30 mg/kg) given systemically reduced alcohol-only drinking in both (**A**) females and (**B**) males. (**C**,**D**) Systemic SB at this dose did not reduce concurrent water intake in either sex. *p < 0.05; **p < 0.01.

Previous studies have shown that a high systemic dose of SB (30 mg/kg) can reduce alcohol drinking in female mice^49^, although some have questioned the specificity of this dose (discussed in^21^). Thus, we examined the impact of this higher SB dose on female and male alcohol-only binge intake. 30 mg/kg systemic SB reduced binge alcohol drinking in both females (Fig. 2A, *n* = 12, *p* = 0.0068, Wilcoxon) and males (Fig. 2B, *n* = 12, *p* = 0.0122, Wilcoxon). A two-way ANOVA found a significant effect of treatment (F_1,22_ = 23.546, *p* < 0.001) but no sex-treatment interaction (F_1,22_ = 0.292, *p* = 0.594). However, this higher SB dose did not alter concurrent water intake in either sex (female: Fig. 2C, *p* = 0.3750: males: Fig. 2D, *p* = 0.2754; Wilcoxon). The decrease in intake with SB 30 mg/kg did not differ between females and males (female: − 34.9 ± 11%, male: − 26.8 ± 12%, intake level during SB exposure relative to vehicle; *p* = 0.7553 between sexes Mann–Whitney). Thus, alcohol-only binge intake in females can be regulated by Ox1Rs, in agreement with previous findings^49^, but our findings above suggest that this did not occur through shell Ox1Rs (Fig. 1A,B), unlike in males^29,43^.

**Figure 3:**
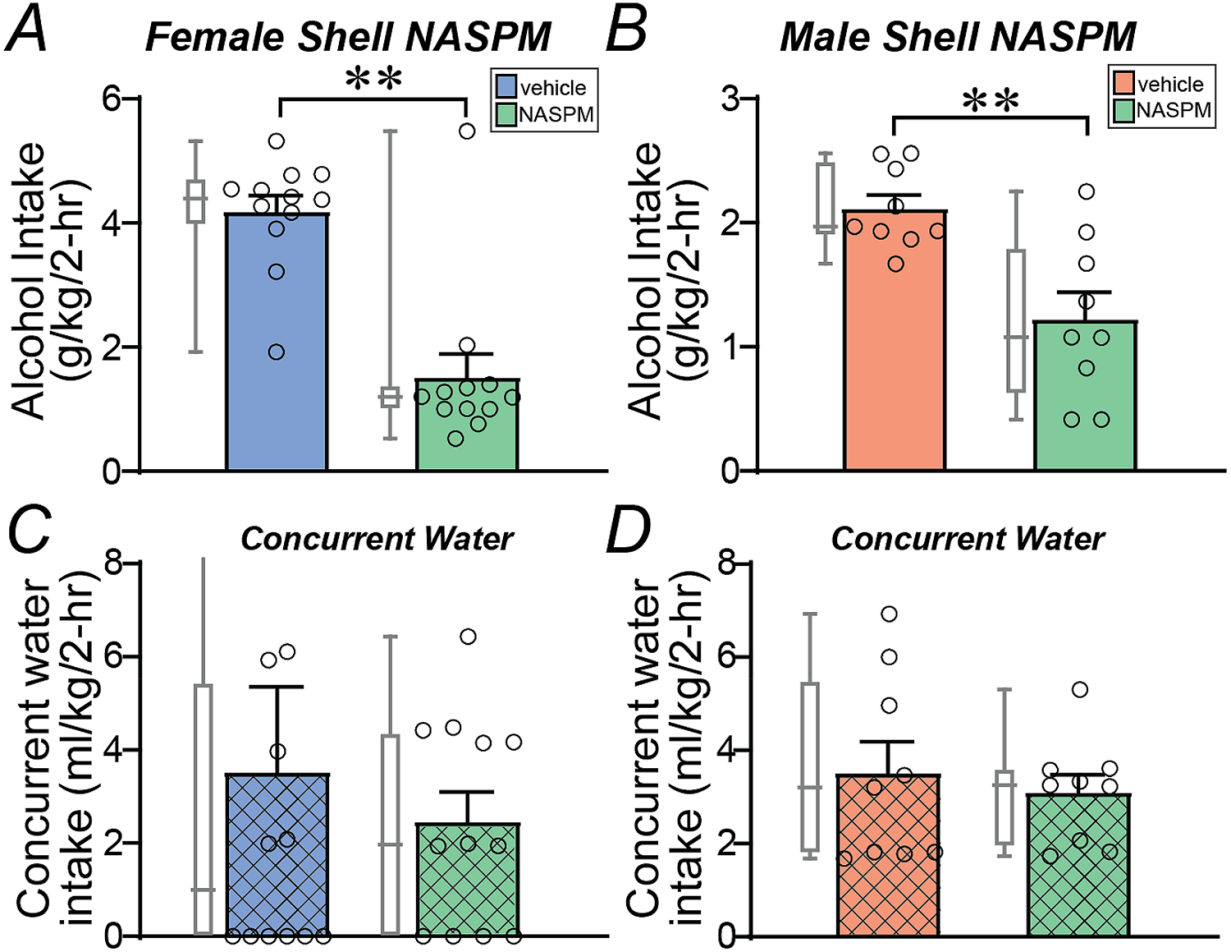
Shell CP-AMPAR inhibition reduced alcohol binging in both sexes. (**A**,**B**) Intra-shell infusion of the CP-AMPAR inhibitor NASPM significantly and strongly reduced alcohol-only drinking in both (**A**) females and (**B**) males. (**C**,**D**) Intra-shell NASPM did not reduce concurrent water intake in either sex. One data point for female concurrent water consumption in (**C**) was 22.2 ml/kg, not shown on graph. **p < 0.01.

To examine whether more general shell activity might be critical for female alcohol binging, we examined whether inhibition of calcium-permeable AMPARs (CP-AMPARs) in shell would suppress binge intake. A number of studies have shown that a variety of alcohol drinking models induce expression of CP-AMPARs in the shell^22,51–53^, with unpublished findings suggesting their promotion of alcohol intake^22^. Thus, we tested whether intra-shell infusion of NASPM (20 µg/side), a widely used and validated CP-AMPAR blocker^22^ (see “Material and methods”), could reduce binge intake. This intracranial NASPM dose was used in many studies^62–65^, and this and higher doses within the NAcb decrease cocaine seeking, but have no effect on cocaine self-administration, sucrose seeking^62,65^ or behavioral flexibility^66^. We found that NASPM strongly and significantly reduced alcohol intake in both females (Fig. 3A, *n* = 12, *t*_11_ = 6.344, *p* < 0.0001) and males (Fig. 3B *n* = 9, *t*_8_ = 4.4, *p* = 0.0023). In strong contrast, intra-shell NASPM did not reduce concurrent water intake in females (Fig. 3C, p > 0.9999, Wilcoxon) nor males (Fig. 3D, *t*_8_ = 0.4294, *p* = 0.6789), suggesting a specific effect on alcohol consumption. A two-way ANOVA found a significant effect of treatment (F_1,19_ = 47.063, *p* < 0.001) and sex-treatment interaction (F_1,19_ = 11.726, *p* = 0.003). Also, while there was a trend for a stronger NASPM effect in females for alcohol drinking (female: 62.8 ± 8.3% decrease in intake; male: 42.5 ± 8.8% decrease in intake), this was not significant (*p* = 0.0955, Mann–Whitney test). Thus, although males but not females required shell Ox1Rs for binge alcohol drinking, shell activity through CP-AMPARs was essential for promoting binge intake in both sexes.

**Figure 4:**
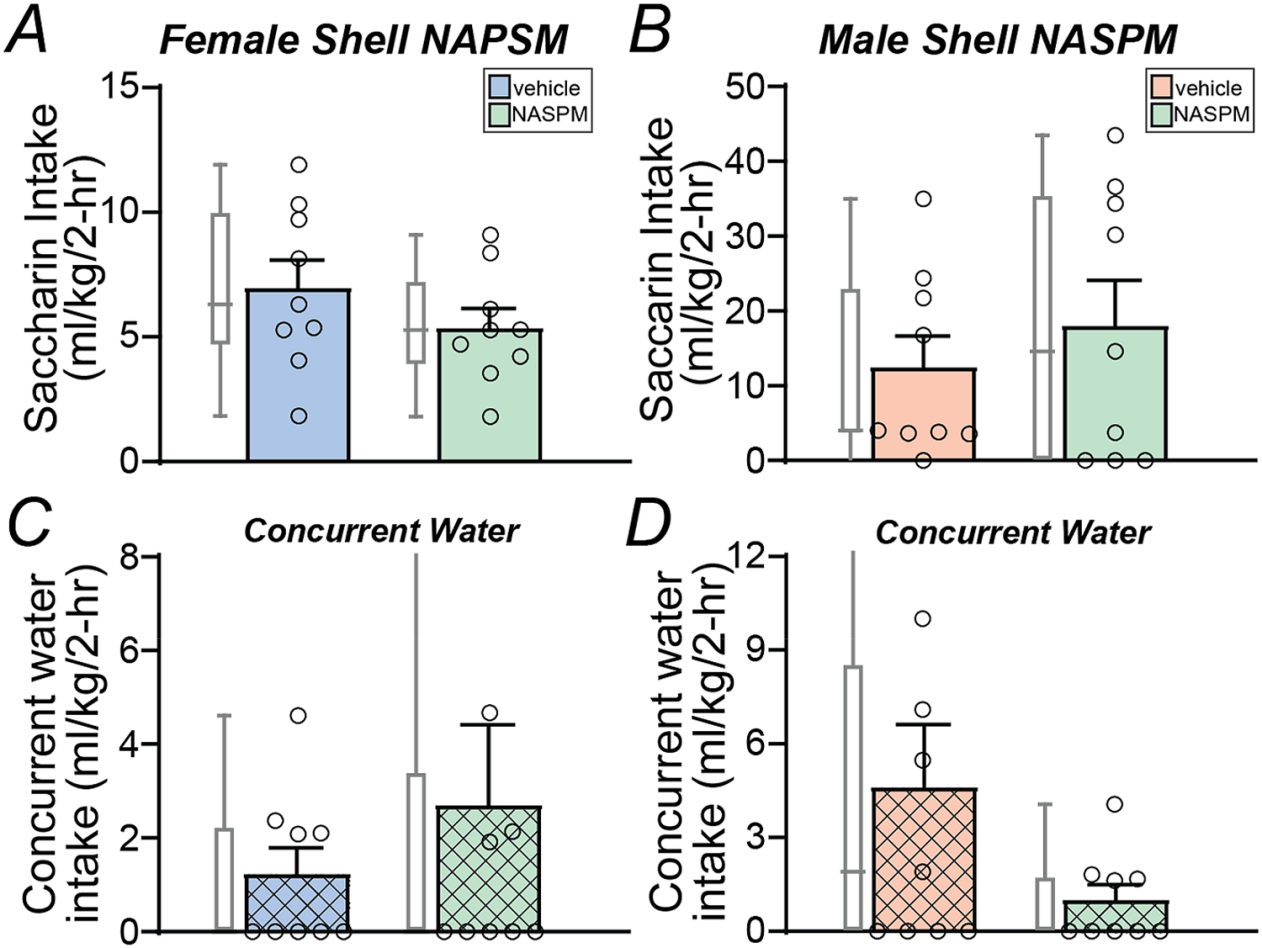
Shell CP-AMPAR inhibition did not reduce saccharin intake in either sex. (**A**,**B**) Intra-shell infusion of NASPM did not reduce saccharin intake in either sex. (**C**,**D**) Intra-shell NASPM did not reduce concurrent water intake in either sex, although a trend is seen in males. One vehicle data point in (**C**) was 15.7 ml/kg, and one NASPM in (**D**) was 17.1 ml/kg, not shown on graphs. Saccharin intake was not different across sexes (*p* > 0.9 Mann–Whitney).

To better understand the specificity of shell CP-AMPAR inhibition on consummatory behavior, we next examined whether NASPM in the shell could alter saccharin intake. However, unlike alcohol drinking, shell NAPSM did not alter saccharin drinking in females (Fig. 4A, *n* = 9, *t*_8_ = 1.677, *p* = 0.1320) or males (Fig. 4B, *n* = 9, *p* = 0.3008, Wilcoxon), nor did it alter concurrent water consumption (females: Fig. 4C, *p* = 0.8125; males: Fig. 4D, *p* = 0.1953; Wilcoxon). Together, these results strongly suggest that shell CP-AMPARs strongly and selectively reduced binge alcohol intake in both female and male mice.

**Figure 5:**
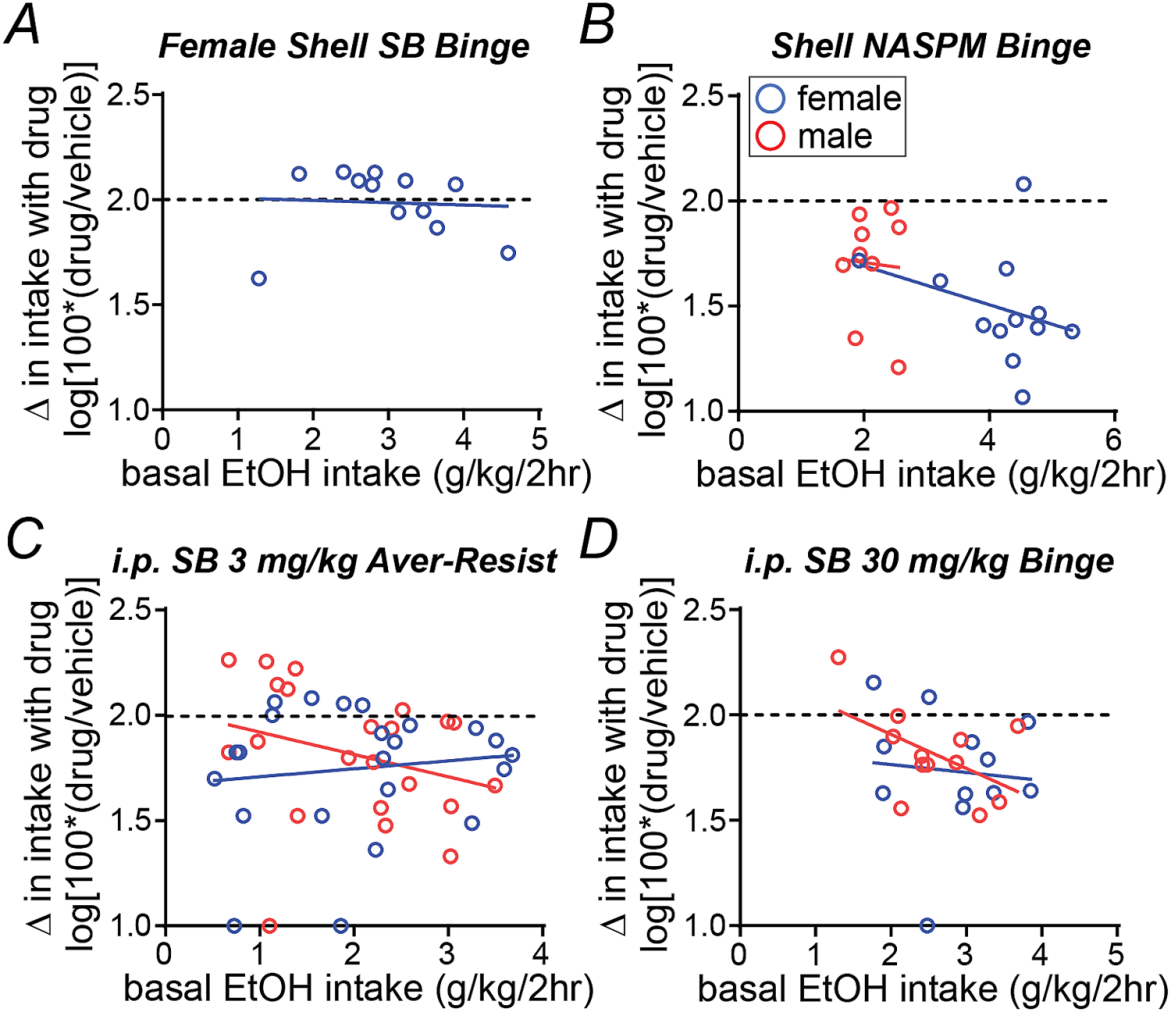
The impact of Ox1R or CP-AMPAR inhibition was minimally related to basal alcohol intake levels across individual mice. For theses analyses, basal intake in each mouse was determined from vehicle injection days, and the change in drinking with drug was determined by log transforming the percent change in drinking (see “Material and methods”). (**A**–**D**) Basal intake was not correlated with change in drinking after (**A**) shell SB infusion in females, (**B**) shell NASPM infusion in either sex, (**C**) systemic 3 mg/kg SB in either sex (quinine-resistant intake), or (**D**) systemic 30 mg/kg SB infusion in either sex.

We previously found that shell Ox1Rs are particularly important for driving binge intake in higher-drinking male mice^43^, suggesting that a lack of overall effect of shell SB in females might mask individual differences. However, there was no significant relation between basal alcohol-only intake level (determined from vehicle injection test days) and the change in drinking with shell SB in females (Fig. 5A, *p* = 0.6129, R^2^ = 0.0266). Furthermore, the impact of shell NASPM was not related to basal drinking level in males (*p* = 0.8147, R^2^ = 0.0084) or females (*p* = 0.2739, R^2^ = 0.0267) (Fig. 5B), suggesting an impact across all individuals (and unlike shell Ox1R inhibition in males which primarily impacts higher drinkers^43^). We also note that the 3 mg/kg SB impact on quinine-resistant drinking was not related to basal intake level in females (*p* = 0.5542, R^2^ = 0.0161) or males (*p* = 0.1977, R^2^ = 0.0815) (Fig. 5C), and that there was no relation between basal intake and 30 mg/kg SB effects on alcohol intake in either sex (male: *p* = 0.0846, R^2^ = 0.2682; female: *p* = 0.7817, R^2^ = 0.0080) (Fig. 5D). Taken together, our results suggest that NASPM in the shell reduced alcohol-only binge drinking across all individuals of both sexes, and that the lack of impact of shell SB on female binge intake was unlikely due to differences in SB effects across individuals.

## Discussion

Binge alcohol drinking, with excessive levels of intake, is a potent and pernicious obstacle to treating AUD, and heavy drinking individuals are responsible for much of the considerable personal and social harm of AUD^1–6^. In addition, problem drinking in females has risen dramatically across recent years^11–13^, and thus it is critical to uncover mechanistic differences across the sexes in order to best develop effective therapies to treat AUD in females versus males. Our previous work has identified the importance of shell Ox1Rs and their role in promoting alcohol intake in higher-drinking male mice^29,43^. Given this and the observation that female mice drank more than males overall, it was originally predicted that females would be more dependent on shell Ox1Rs. However, here we show that shell Ox1Rs were not needed to promote alcohol-only drinking in female mice. However, aversion-resistant alcohol drinking was similarly reduced by lower doses of systemic Ox1R inhibition in males and females, showing that Ox1Rs can regulate at least some forms of alcohol intake in females. Similarly, higher systemic SB doses also reduced alcohol-only drinking in both sexes (see below). Furthermore, our findings indicate the critical importance of shell CP-AMPARs for supporting binge alcohol intake in both males and females, suggesting that shell mediation of binging was important for alcohol drinking in both sexes, but through complementary mechanisms. Also, this role of shell CP-AMPARs was specific for alcohol, as CP-AMPAR inhibition had no effect on concurrent water intake or saccharin drinking in either sex. Finally, blood alcohol measures confirm that, on average, male and female mice reached binge-level alcohol intake (Suppl. Fig. 2). Taken together, our results suggest that the shell was critical for promoting alcohol-only binge drinking in both sexes (e.g. through CP-AMPARs), but that shell Ox1Rs were important in males but not females (discussed further below).

One central finding of the present study is that shell Ox1Rs were not important for supporting female binge alcohol drinking, while our previous work underscores the critical role of shell Ox1Rs in male alcohol intake^29,43^. Orexin has long been recognized as an important regulator of alcohol behaviors^21,38,39^, with full consideration beyond the scope of this work. Previous studies have assessed the relative impact of OxRs across females and males, and the different impact of shell Ox1Rs in females and males could reflect differential function or expression across the sexes. Sex and estrous differences in orexin and OxRs have been observed in the hypothalamus^76^, but not in cortical and other subcortical areas^76–78^. There are also no sex differences in OxR regulation of morphine mesolimbic activation, sucrose intake, or stress-induced cocaine seeking^79–81^. Further, systemic Ox1R blockers reduce 2-bottle choice alcohol intake in both sexes^45,49^. However, Ox1R inhibition impacts operant-based alcohol intake in male alcohol-preferring P-rats^82^ with only a trend in female P-rats^49^. In outbred rats, Ox1R inhibition reduces alcohol seeking in males^38^, with effects in females only if alcohol is present^83^. Considerable future work will be required to disambiguate the number of possible mechanisms that might underlie sex differences in shell Ox1R regulation, including differences in Ox1R expression that might vary across cell types, differing release of orexin, potential orexin interactions with other neuromodulator systems (e.g. ^84^), and other possibilities. In addition, it would have been useful to examine another behavior in the present studies to confirm that shell SB used in female alcohol-drinking studies was functional. Importantly, shell SB experiments in females were done concurrently with male SB shell experiments^43^, giving confidence that shell SB was functional and able to modulate alcohol drinking under some conditions (males drinking under DID or intermittent access or LDA, the latter method used for the present studies)^43^. Nonetheless, it would be valuable in future studies to understand conditions under which female shell Ox1Rs are important for behavior, e.g. reinstatement of morphine CPP^61^.

We note that shell Ox1Rs were not required for female alcohol-only intake, unlike in males, but systemic inhibition of Ox1Rs utilizing lower doses of SB reduced compulsion-like alcohol drinking in both females and males (for the latter, see also^48^). In this regard, it is also interesting that, while males and females both exhibit reinstatement for cocaine and sucrose^79,81^, Ox1R inhibitors reduce both cue- and stress-related reinstatement in males, but only stress- and not cue-induced reinstatement in females^79,81^. Taken together, these findings might lead to the speculation that Ox1Rs in females are important for more stress-related behaviors (stress-induced reinstatement and compulsion-like intake) but are not involved in more basic behaviors (binge drinking, cued reinstatement), while Ox1Rs in males would be important for a broader range of motivated behaviors. However, across a large sample of mice, we find that shell Ox1Rs are primarily important for alcohol drinking in higher-drinking males, with lesser importance in moderate bingers^43^, perhaps consistent with previous findings that systemic inhibition of Ox1Rs reduces alcohol drinking in dependent but not non-dependent mice^50^ and in higher- but not lower-drinking rats and mice^44–46^. However, considerable additional studies would be required to address these sex-, basal-intake-, and challenge-related possibilities. We also note that a higher dose of SB, given systemically, did inhibit alcohol-only consumption in both females and males, in agreement with some previous studies^45,49^, although some have questioned the specificity of this high dose (see^21^); the brain site of these effects also remain open, since, in addition to Ox1Rs in the shell and medial prefrontal cortex^29^, Ox1Rs in central amygdala and ventral tegmental area also promote binge alcohol drinking in mice^85^. We also note that we did not perform a dose–response of systemic SB here. Our previous work performed a dose–response in male C57 mice for both alcohol-only LDA drinking and aversion-resistant drinking^48^, and our alcohol-only data in males show a similar dose–response for female C57 mice alcohol-only drinking in Anderson et al.^49^. Thus, here we used a lower SB concentration (3 mg/kg) for aversion-resistant drinking, which in males impacts quinine-resistant but not alcohol-only drinking^48^ and does not impact alcohol-only intake in females^49^. In addition, we tested 30 mg/kg SB for effects on alcohol-only drinking, since this is the only dose to significantly reduce alcohol-only drinking in female C57 mice in^49^, and this dose is very widely used as an effective dose with some selectivity of behavioral impact^21^.

As we reviewed elsewhere^21^, there are two endogenous peptides, orexinA and orexinB, and two orexin receptors, which can mediate orexinergic signaling. Orexin-2-receptors (Ox2Rs) have been related more to sleep, arousal and stress, while Ox1Rs are more related to addiction, reward and motivation. However, there clearly can be crossover, e.g. where Ox2Rs regulate operant responding for alcohol^86^. We found in mice that Ox2Rs in shell do not contribute to male binge-like alcohol drinking^29^, while systemic inhibition of Ox2Rs does not regulate male aversion-resistant intake^48^. Nonetheless, there are clear sex differences in regulation of stress and alcohol behaviors^14,87^, and thus future studies should examine whether Ox2Rs might play a role in females different from males. Further, while downstream signaling molecules of orexin in neurons are only partially understood, it would be valuable to identify orexin receptor linkage to protein kinase C and other orexin-modulated systems (e.g.^88^).

While shell Ox1Rs were not needed for female alcohol intake, inhibition of CP-AMPARs in the shell with the widely utilized NASPM significantly and strongly reduced alcohol intake in both males and females. Thus, shell CP-AMPARs were potent promoters of alcohol binging in both sexes. CP-AMPARs in the shell are apparent in relation to various challenges, and inhibition of shell CP-AMPARs can reduce several addiction-related behaviors (reviewed in^22^). Furthermore, our findings are consistent with previous studies showing^51–53^ and suggesting^89,90^ that several forms of alcohol exposure lead to the appearance of CP-AMPARs in the shell. It would be interesting in future studies to determine the nature of any molecular changes in CP-AMPARs in female compared to male alcohol-drinking mice. Importantly, while intra-shell inhibition of CP-AMPARs potently reduced binge alcohol intake in both sexes, these effects were specific for alcohol, since intra-shell NASPM did not significantly reduce concurrent water intake during alcohol drinking, nor did it decrease saccharin drinking (tested in a different cohort). Thus, our results support the importance of the shell for promoting binge alcohol drinking across sexes through CP-AMPARs.

We note that we did not assess the possible impact of estrous cycle on drinking in female mice. While hormonal changes can impact drinking levels under some conditions (e.g.^91^), several studies find that the estrous cycle can have limited influence on addiction-related behavior once established^14,18,92,93^, including binge-like drinking in female mice^91^ or compulsion-like behaviors for alcohol^19^. Thus, the findings of Satta and colleagues^91^ in particular suggest that female C57 mouse alcohol drinking does not vary across the intact estrous cycle. Nonetheless, future studies could examine how the influence of specific shell receptor types on alcohol drinking might vary across the estrous cycle.

Taken together, our findings here and elsewhere^29,43^ support the critical importance of the shell in driving binge alcohol drinking in female and male mice. However, there are important similarities and differences in underlying mechanisms, since CP-AMPARs were crucial in both sexes, while shell Ox1Rs are only required in higher-drinking male mice^43^. However, lower systemic doses of the Ox1R blocker SB inhibited compulsion-like alcohol drinking in both sexes, demonstrating that Ox1Rs can regulate at least some forms of pathological alcohol drinking in females similar to males. Since binge alcohol drinking is a strong contributor to the harms of human drinking^1–6^, and problem drinking in females has risen dramatically across recent years^11–13^, our findings uncovering mechanistic differences across the sexes may help to develop more effective sex-selective and -general therapies to treat AUD.

## Supplementary Information

Supplementary Information
