# Supplementary material for: Differential importance of nucleus accumbens Ox1Rs and AMPARs for female and male mouse binge alcohol drinking
Source: Sci Rep. 2021 Jan 8;11:231. doi: 10.1038/s41598-020-79935-2 (PMC7794293; doi:10.1038/s41598-020-79935-2)

Claudina Kwok, Kelly Lei, Vincent Pedrozo, Lexy Anderson, Shahbaj Ghotra, Margaret Walsh[b], Laura Li, JiHwan Yu, and Frederic Woodward Hopf

Differential importance of nucleus accumbens Ox1Rs and AMPARs for female and male mouse binge alcohol drinking

Supplemental Figure 1. Histology. Bottom of cannulae are shown, with injector 0.3 mm below. **(A)** Female Shell SB (Fig.1A), **(B)** Female Shell NASPM for alcohol (Fig.2A), **(C)** Male Shell NASPM for alcohol (Fig.2B), **(D)** Female Shell NASPM for saccharin (Fig.3A), **(E)** Male Shell NASPM for saccharin (Fig.3B). **(F)** Two representative micrographs. Alc: alcohol; Sac: saccharin.

Supplemental Figure 2. Blood alcohol measurements. **(A)** In both **(A,B)** females and **(B)** males, blood alcohol significantly correlated, and on average were above 80 mg% indicating binge levels of intake (see Results). Alcoh: alcohol; BAC: blood alcohol concentration. * $p<0.05$; ** $p<0.01$.

Kwok et al., Figure S1

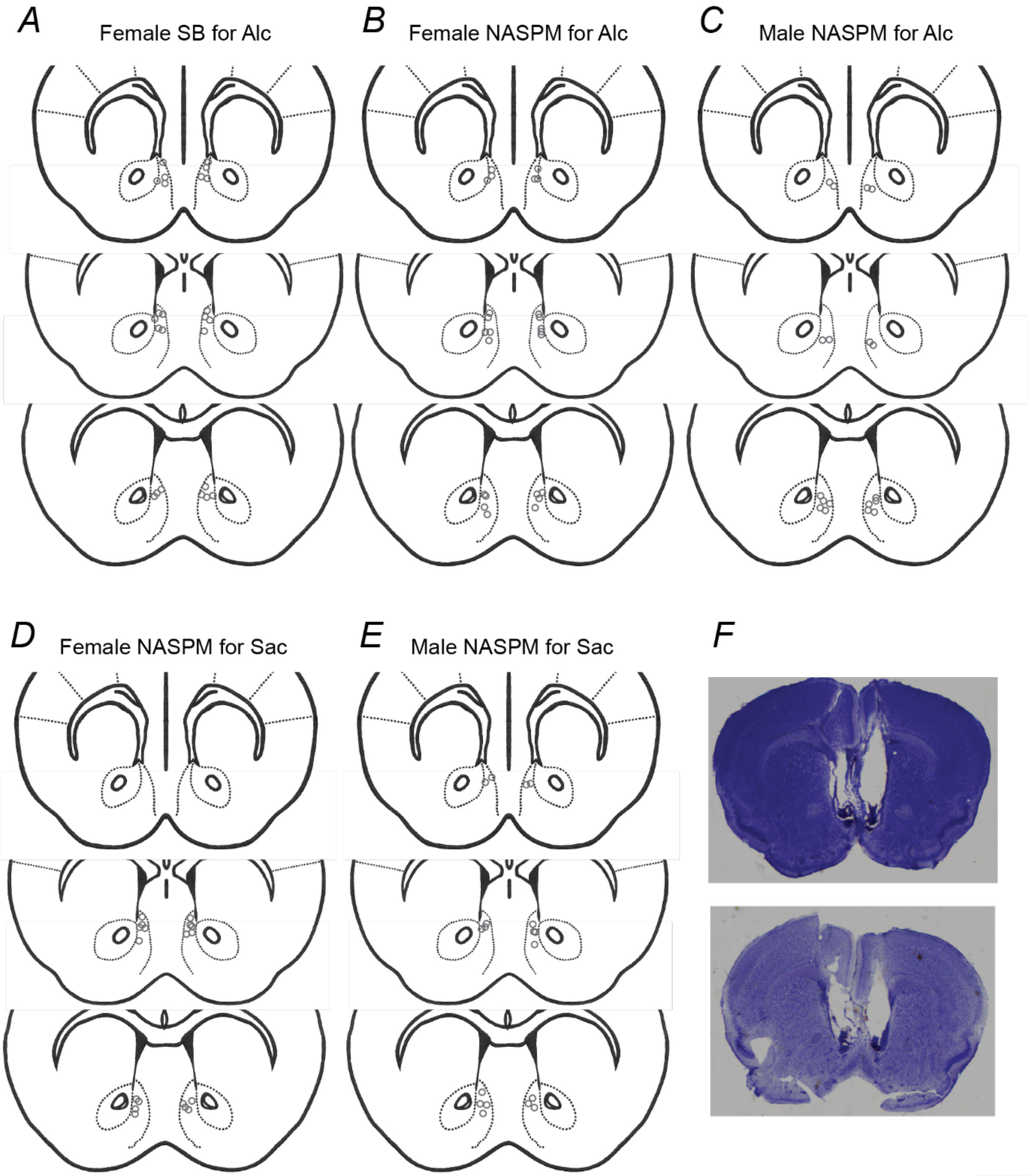

Kwok et al., Figure S2

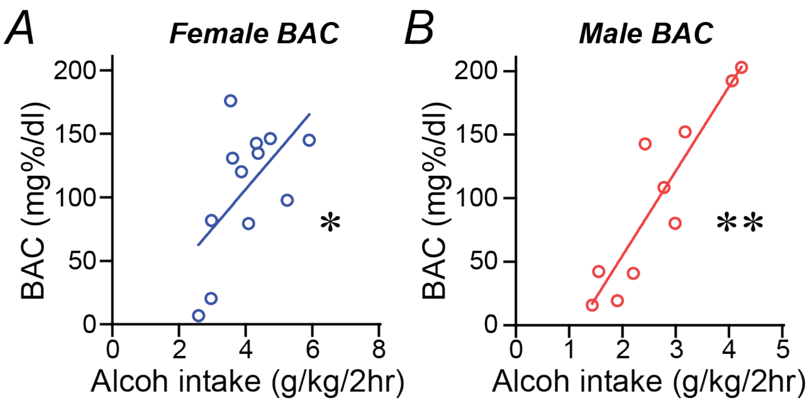

Supplement: Supplementary file 1 — Supplementary Information [file 41598_2020_79935_MOESM1_ESM.pdf]
